# Supplementary figures and images for: lcc: an R package to estimate the concordance correlation, Pearson correlation and accuracy over time
Source: PeerJ. 2020 Sep 17;8:e9850. doi: 10.7717/peerj.9850 (PMC7502249; doi:10.7717/peerj.9850)

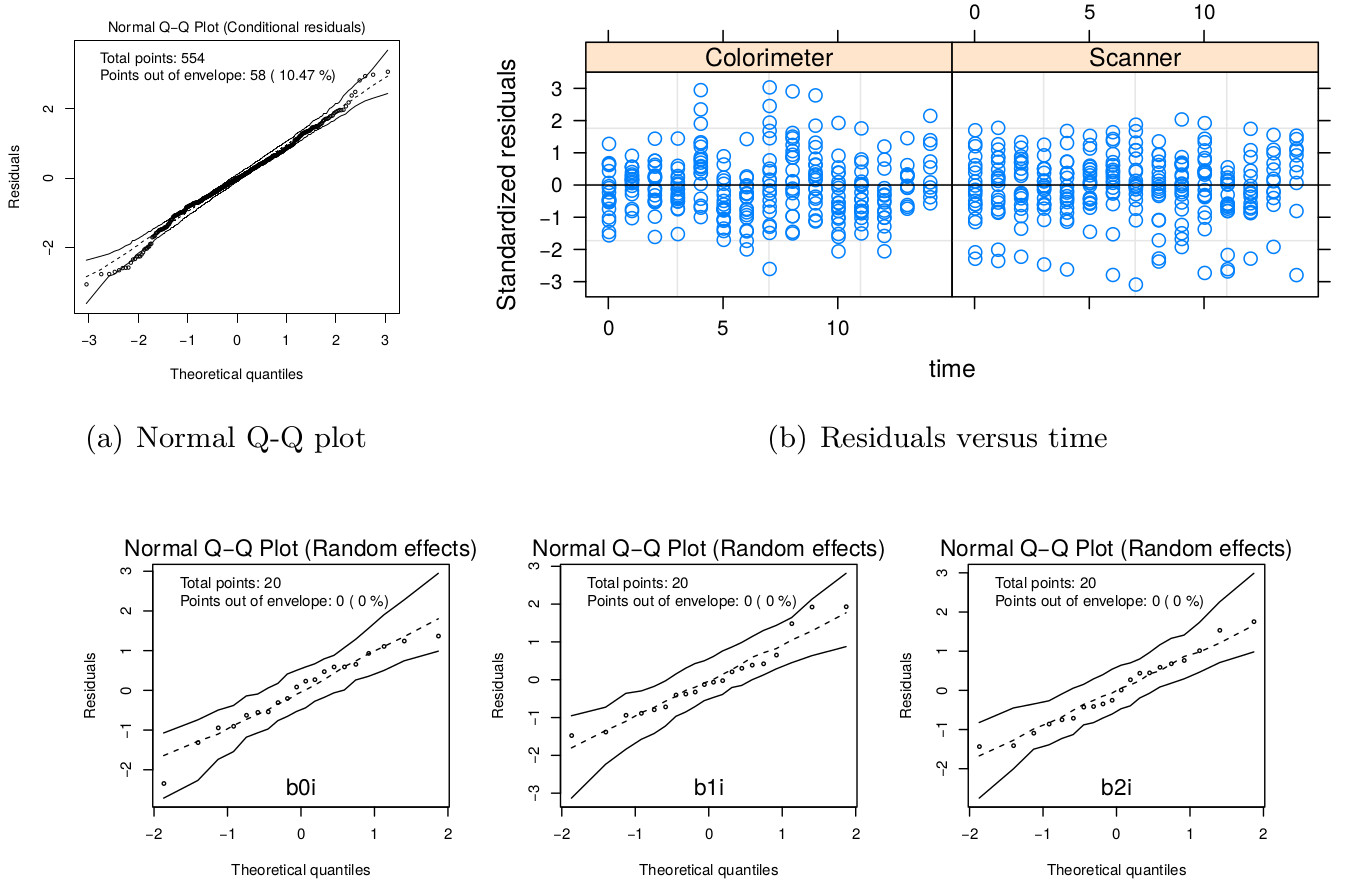

Supplement: Supplemental Information 1 — (A) Normal plot of within-group standardized residuals, (B) plot of standardized residuals versus time for the m1 fitted model object; and (C) normal Q-Q plot with 95% simulate envelop for random effects [file peerj-08-9850-s001.jpeg]

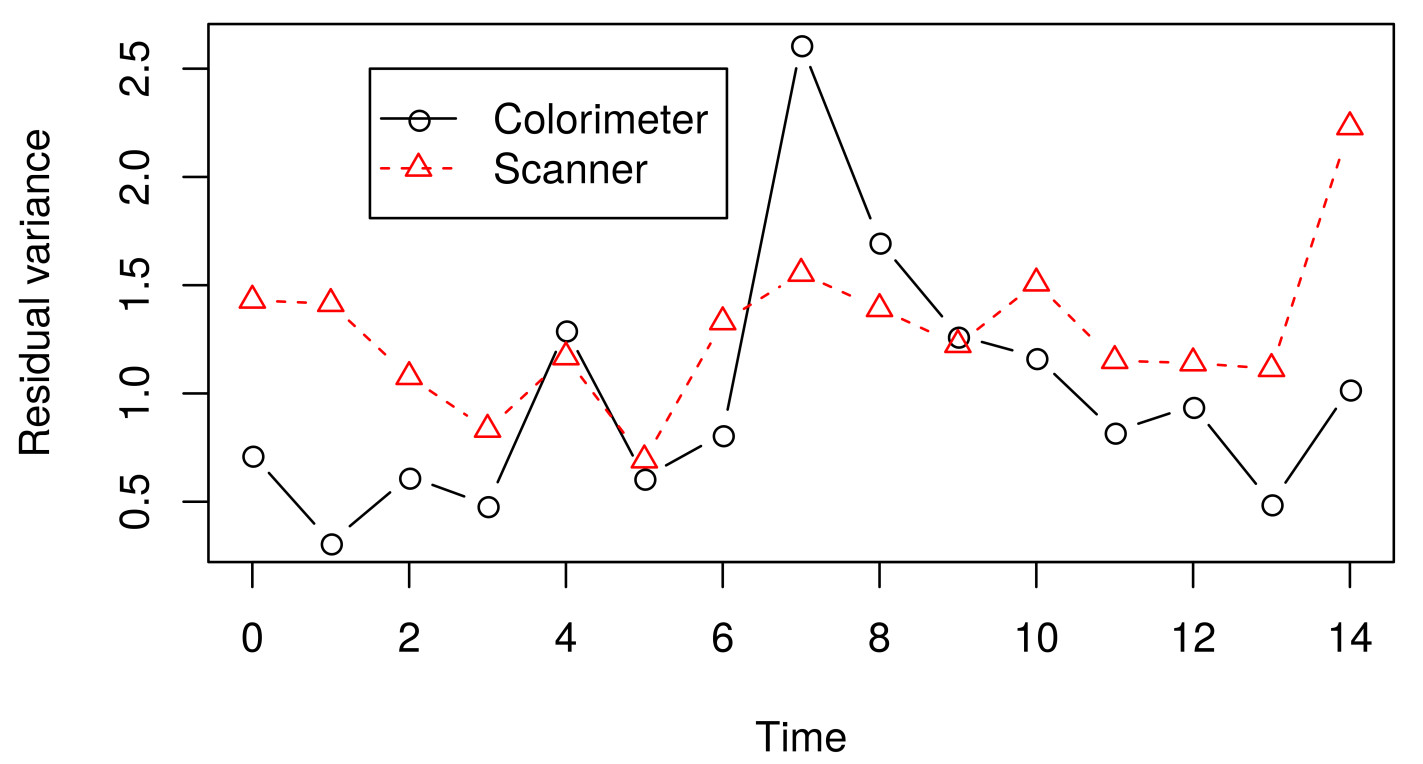

Supplement: Supplemental Information 2 — Residual variance for residuals within method (colorimeter or scanner) over time [file peerj-08-9850-s002.jpeg]

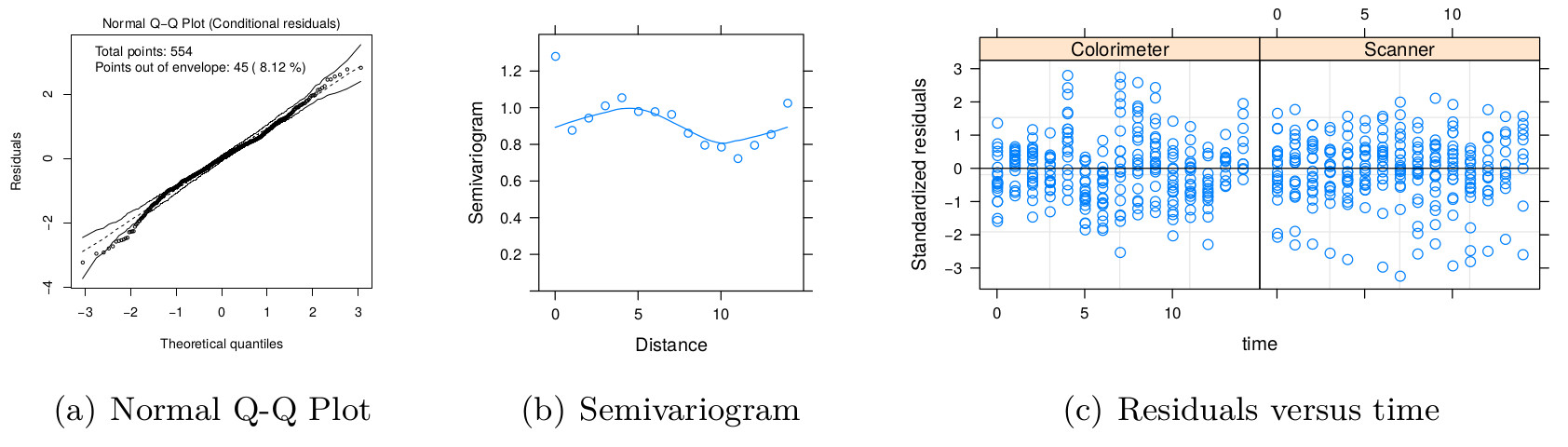

Supplement: Supplemental Information 3 — (A) Normal plot of within-group standardized residuals, (B) sample semivariogram of the standardized residuals, and (C) plot of standardized residuals versus time for the m.hue.3 fitted model object [file peerj-08-9850-s003.jpeg]
