# Supplementary material for: lcc: an R package to estimate the concordance correlation, Pearson correlation and accuracy over time
Source: PeerJ. 2020 Sep 17;8:e9850. doi: 10.7717/peerj.9850 (PMC7502249; doi:10.7717/peerj.9850)
Supplement: Supplemental Information 7 [file peerj-08-9850-s007.pdf]

# Time computations

May 19, 2020

The time computations to construct the confidence intervals based on 10,000 bootstrap samples for each model fitted for all examples in the paper are reported in Table 1, those analysis were executed on Dell Inspiron 17 7000 with $10^{\text{th}}$ Generation Intel® Core™ i7 processor, 1.80GHz × 4 processor speed, 16GB random access memory (RAM) plus 20GB of swap space, 64-bit integers, and the platform used is a Linux Mint 19.2 Cinnamon system version 5.2.2-050202-generic. Furthermore, all code was executed in the GNU Emacs Editor with R version 3.6.1. Table 1 shows that when the `optim` method is used the computational time is

Table 1: Time to construct the confidence interval based on 10.000 bootstrap samples for each fitted model using a specific number of cores (NumCore)

| Data | Model | Time | NumCore |
|---|---|---|---|
| Body fat | `m.bfat.1` | 18.02 | 1 |
| | `m.bfat.2` | 12.66 | 1 |
| Hue | `m.hue.2` | 51.43 | 1 |
| | `m.hue.3` | 36.33 | 1 |
| Blood draw | `m.bw.2` | 390.94 | 1 |
| | `m.bw.2` | 257.70 | 3 |

reduced in 5.36 minutes for the Body fat data and 15.1 for the Hue data analysis in relation to `nlminb` method. However, as the `nlminb` is the default method into `nlme` package, we decided to continuous using the same default. In addition, for the mode `m.bw.2` we observed a time reduction of 133.24 minutes when we increase the number of cores from 1 to 3.
