# Supplementary material for: lcc: an R package to estimate the concordance correlation, Pearson correlation and accuracy over time
Source: PeerJ. 2020 Sep 17;8:e9850. doi: 10.7717/peerj.9850 (PMC7502249; doi:10.7717/peerj.9850)
Supplement: Supplemental Information 8 [file peerj-08-9850-s008.pdf]

Comparison between the `lcc` and `cccrm` packages

February 9, 2020

In this section we review the `cccrm` `R` package proposed by [2] to estimate the CCC for repeated or non-repeated measures data and discuss how this package differs from our package `lcc`. We can use the `ccclon` and `ccclonw` functions to estimate the CCC for repeated measures (CCC_{rm}) and these were the first ones of this type available for `R`. Both functions estimate the CCC based on variance components of a mixed-effects model for longitudinal data, however, the difference between them is that `ccclonw` uses a non-negative definite matrix of weights between different repeated measurements [1].

These functions have been introduced to produce a value that summarizes the interchangeability between methods in relation to all of their measurements, rather than by modeling the agreement as a function over time [1]. On the other hand, the `lcc` function in package `lcc` was developed to capture changes in the extent of the agreement profile between methods. Furthermore, `lcc` also provides estimates and confidence intervals for LPC and LA that are important statistics to make inferences on both the precision and accuracy of the measurements, respectively, and how they affect the LCC at different time points.

We now estimate the CCC_{rm}, using package `cccrm`, between the scanner and the colorimeter measurements of the papaya peel hue dataset:

```
R> library(cccrm)
R> data(hue, package = "lcc")
R> ccclon(hue,"H_mean","Fruit","Time","Method")
CCC estimated by variance components:
CCC         LL CI 95%     UL CI 95%      SE CCC
0.83767698    0.72520268    0.90660774    0.04486742
```

The estimate of the CCC_{rm} shows a moderate/poor agreement between the methods, suggesting that the digital image analysis of the equatorial peel region should be not used to compute the mean hue. However, suppose that the researcher had stipulated the following condition: "we would only take measurements on the equatorial region using a colorimeter

if the lower band of confidence interval is greater than or equal to 0.90". Thus, based on the lower band of the CCC_{rm} (0.725), the researcher should not use the colorimeter to compute the mean hue. On the other hand, the lower band of the LCC (Figure 8 of the paper) indicates that the papaya's equatorial region can be adequately sampled through four equidistant points using a colorimeter from the ninth day. Clearly, this conclusion is only valid under the same experimental conditions.

REFERENCES

[1] Josep L Carrasco, Tonya S King, and Vernon M Chinchilli. The concordance correlation coefficient for repeated measures estimated by variance components. *Journal of Biopharmaceutical Statistics*, 19(1):90–105, 2009.

[2] Josep L. Carrasco, Brenda R. Phillips, Josep Puig-Martinez, Tonya S. King, and Vernon M. Chinchilli. Estimation of the concordance correlation coefficient for repeated measures using SAS and R. *Computer Methods and Programs in Biomedicine*, 109:293–304, 2013.
